# Effects of ultrasound and microwave pretreatments on hydrodistillation extraction of essential oils from Kumquat peel

**DOI:** 10.1002/fsn3.2073

**Published:** 2021-03-27

**Authors:** Fen Yu, Na Wan, Qin Zheng, Yuanhui Li, Ming Yang, Zhenfeng Wu

**Affiliations:** ^1^ Key Laboratory of Modern Preparation of TCM Ministry of Education Jiangxi University of Traditional Chinese Medicine Nanchang China

**Keywords:** essential oil, kinetics, microwave pretreatment, quality, ultrasound pretreatment

## Abstract

Main objectives of this work were to investigate the influences of ultrasound pretreatment (UP) and microwave pretreatment (MP) on extraction kinetics, chemical composition, and antioxidant activity of Kumquat peel essential oil (EO) obtained by hydrodistillation extraction (HDE). The effects of ultrasound power and processing time, and microwave power and processing time were evaluated. As compared with HDE individually, UP and MP decreased the extraction time, increased the yield and DPPH radical‐scavenging activity but did not noticeably affect chemical composition of the EO. For UP and MP, the highest EO yield was obtained when the ultrasonic power and processing time, and microwave power and processing time were 210 W and 30 min, 300 W and 6 min, respectively. In comparison with MP, UP gave a higher yield and DPPH radical‐scavenging activity of the EO. Overall, UP and MP are promising techniques for HDE of EO from kumquat peel.

## Graphical abstract

Main objectives of this work were to investigate the influences of ultrasound pretreatment (UP) and microwave pretreatment (MP) on extraction kinetics, chemical composition and antioxidant activity of Kumquat peel essential oil (EO) obtained by hydrodistillation extraction (HDE). As compared with HDE individually, UP and MP decreased the extraction time, increased the yield and DPPH radical scavenging activity but did not noticeably affect chemical composition of the EO. In comparison with MP, UP gave a higher yield and DPPH radical scavenging activity of the EO. Overall, UP and MP are promising techniques for HDE of EO from kumquat peel.

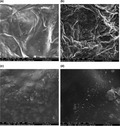

## 1 INTRODUCTION

Kumquat (*Fortunella margarita* Swingle), an important genus closely related to Citrus of the Rutaceae family, is widely cultivated in Asia‐Pacific region. Kumquat fruit is frequently consumed as one of fruits all over the world (Nouri & Shafaghatlonbar, 2016). Unlike the fruits of other citrus species, kumquat fruit is usually eaten as a whole fruit together with the peel. It is well known that the peel of kumquat fruit is rich in essential oil which is an important factor affecting the taste of the fruit and is widely used in food and pharmaceutical industries owing to its various functional properties, such as an attractive aroma, a repellant agent against insects and animals, and antioxidant and antimicrobial activities (Wang et al., 2012).

Extraction is an indispensable step for obtaining EO from natural plant. The common industrial method for EO is hydrodistillation extraction (HDE). However, HDE is a time‐consuming and low‐efficiency process (Périno‐Issartier et al., 2013). Up to now, many attempts have been made to improve the HDE efficiency by using energy‐intensive techniques, such as ultrasound pretreatment (UP) and microwave pretreatment (MP). Ultrasound utilizes the mechanical, cavitation, and thermal effects to destroy the cell walls of the plant matrix, which accelerates the release of contents into the extraction medium (Chemat et al., 2011). Therefore, UP can reduce the extraction time and improve the extraction efficiency (Dimaki et al., 2017; Taticchi et al., 2019). Microwave uses electromagnetic waves that pass through rapidly and dissipate volumetrically inside the medium, causing fast heat transfer and changes in the cell structure (Veggi et al., 2012). Hence, the target compounds can be rapidly transferred from the plant matrix to the solvent (Liu et al., 2018).

However, to the best of our knowledge, there is no reported study for the application of UP and MP to HDE of EO from kumquat fruit. In addition, few reports focus on comparing the effect of UP and MP on the HDE of EO. Thus, the main objective of this research was to evaluate the effects of UP and MP on the dependent variables of extraction kinetics and quality attributes (chemical composition and antioxidant capacity) of kumquat peel EO, and the influence of processing parameters include ultrasonic power, ultrasonic time and microwave power, microwave time were investigated.

## 2 MATERIALS AND METHODS

### 2.1 Chemical reagents

Tris buffer (pH = 8.0), DPPH (1,1‐diphenyl‐2‐picrylhydrazyl, 98%, HPLC), NBT (nitro blue tetrazolium) and normal alkane standard solution (C8‐C40) were purchased from Sigma Chemicals Co. All chemicals were of analytical grades and were used without further purification.

### 2.2 Collection and preparation of plant materials

The kumquat was harvested in April 2019, and was collected from the experimental orchard of Jiangxi Agricultural University, located in Jiangxi province, China. The peel was separated and dried in a thermo‐ventilate stove at 40°C for 7 hr, from which a dry matter content near 70% w/w was obtained. The dried material was packed and stored at 4°C.

### 2.3 Hydrodistillation extraction (HDE)

Conventional hydrodistillation was performed with a Clevenger apparatus. Dried kumquat peel (100 g) was added into 800 ml of distilled water. The mixture was distilled for a period of time until no more EO was obtained. The moisture was removed from the EO by adding sodium sulfate anhydrate, and the dried oil was preserved in an amber‐colored vial at 4°C.

### 2.4 Ultrasound pretreatment (UP)

Dried kumquat peel (100 g) was added into 800 ml of distilled water. The ultrasonic power and the ultrasonic treatment time were set to 150, 210, 240, and 270 W and 15, 20, 30, and 40 min, respectively. After the ultrasonic pretreatment, HDE was carried out for a period of time until no more EO was obtained. The subsequent procedure was similar to the HDE method.

### 2.5 Microwave pretreatment (MP)

Dried kumquat peel (100 g) was added to 800 ml of distilled water. The microwave power and the microwave treatment time were set at 200, 300, 500, and 700 W and 3, 6, 10, and 15 min, respectively. After the microwave pretreatment, HDE was carried out for a period of time until no more EO was obtained.

### 2.6 Procedures

#### 2.6.1 Determination of EO Yield

The yield of EO is determined as follows.

(1) Y(%)=V/m×100

where Y (%) is the yield of EO; *V* (mL) is the mean volume of essential oil, and *m* (g) is the mean mass of kumquat peel.

#### 2.6.2 Modeling of extraction kinetics

The extraction kinetics was based on the volume of EO measured at intervals throughout the extraction process. When the EO began to flow out, the volume of the oil was recorded every 30 min. The changes in the EO volume during the three extraction processes were described using a extraction model from the one proposed by (Saidj et al., 2009) Saidj et al. (2009) with small modifications by us. The model is given as follows.

(2) $$V_{t}=a‐e^{\left(V_{1}‐kt\right)}$$

where V*_t_*, V*_1_*, *a,* and *k* denote the moisture content achieved after extracting time *t*, 50% of the maximum amount of oil that can be distilled by the process, and the constant and the rate at which EO are extracted from the medicinal material, respectively.

#### 2.6.3 Gas chromatography–Mass spectrometry analysis

Kumquat peel EO composition was determined using gas chromatography coupled with mass spectrometry (GC–MS) (7890A/5975C, Agilent, USA). An Agilent computerized system comprising a 5,975 gas chromatograph coupled with a 7890A mass spectrometer was used. Gas chromatography analyses were performed with the HP 5,975 gas chromatograph equipped with a FID detector and an HP‐5™ fused silica capillary column (30 m × 0.25 μm × 0.25 μm film thickness) using helium as the carrier gas (1.0 ml/min) at a splitting ratio of 1:20. The injector and detector temperature were 280°C and 250°C, respectively. The oven temperature was initially held at 60°C for 2 min, then linearly increased by 8°C/min until reaching 250°C, and then held for 20 min. Ionization was obtained by electronic impact under a potential of 70 eV, with an ion source temperature of 230°C and the quadrupole temperature of 150°C. The mass spectra were recorded on a selective quadrupolar type Hewlett‐Packard detector model 7890A. Identification of components was mainly based on the comparison of their GC Kovats retention indices (RI), determined with reference to an homologous series of C8–C40 n‐alkanes. GC retention times were also analyzed, and computer matching with the NIST 11 library and comparison of the fragmentation patterns with those reported in the research literature were also performed to ensure accuracy.

#### 2.6.4 Determination of antioxidant activity

##### DPPH radical‐scavenging assay

The DPPH free radical‐scavenging activities of the EO obtained by HDE, UAHE, and MAHE, respectively, were determined using the methods described in the literature (Ma et al., 2012). A 4 ml 0.1 mM DPPH in absolute ethanol solution was mixed with 2 ml of different concentrations of EO (5, 20, 40, 60, 80, 100, 140, 160, and 200 µl/ml). The mixture was incubated for 30 min in the dark at room temperature. Scavenging activity was measured in a spectrometer by monitoring the decrease of absorbance at 517 nm using absolute ethanol as a blank control. Lower absorbance of the reaction mixture indicated higher free radical‐scavenging activity. DPPH radical‐scavenging activity was calculated as Equation 3.

(3) $$\text{SR}\left(\%\right)=\left(A_{0}‐A_{1}\right)/A_{0}\times 100$$

where SR is free radical‐scavenging rate, A_0_ is the absorbance of the control at 30 min, and A_1_ is the absorbance of the sample at 30 min. All samples were analyzed in triplicate.

##### Superoxide anion scavenging activity assay

The superoxide anion radical‐scavenging activity was measured by the method described in the literature (Sunil et al., 2014). The reaction mixture consists of 1 ml of (50 mM) sodium carbonate, 0.4 ml of (24 mM) NBT, and 0.2 ml of 0.1 mM EDTA solutions was added to the test tube and the immediate reading was taken at 560 nm. About 0.4 ml (1 mM) of hydroxylamine hydrochloride was added to initiate the reaction; then, reaction mixture was incubated at 25°C for 15 min and the reduction of NBT was measured at 560 nm. Absorbance was recorded, and the percentage of inhibition was calculated using Equation 3.

##### Hydroxyl radical‐scavenging activity assay

The hydroxyl radical‐scavenging activity was performed by the most commonly used method (Hamasaki et al., 2008). The reaction mixture consisted of 2ml 6 mM FeSO_4_, 1.2 ml 6 mM H_2_O_2,_ and 1 ml sample solutions at different concentrations (5, 20, 40, 60, 80, 100, 140, 160, and 200 µl/ml). The mixed solution was pre‐incubated at 25 ℃ for 10 min and then initiated by the addition of 2 ml 20 mM salicylic acid. The mixture was incubated for 30 min in the dark at room temperature. The absorbance A_1_ was read at 510 nm. Hydroxyl radical‐scavenging activity was then calculated using Equation 3.

#### 2.6.5 Scanning electron micrographs (SEM) observation

Microstructure observations of the raw and the extracted residues were carried out using a SEM (SEM; LEO435VP, England). Samples were dried, fixed, and coated with gold, and then examined under high vacuum condition at a voltage of 10.0 kV (40 µm, 3,000 magnification).

### 2.7 Statistical analyses

All experimental measurements were conducted in triplicate, and the data are expressed as mean ± standard deviation. The data obtained in this study were analyzed by one‐way analysis of variance (ANOVA) using Origin 8.5. Images were processed using Origin 8.5 and GraphPad Prism 8.0.2. Statistical significance was considered at the 5% level (*p* < .05).

## 3 RESULTS AND DISCUSSION

### 3.1 Effect of EO extraction yield

#### 3.1.1 Effect of ultrasonic power and ultrasonic time on the yield

**FIGURE 1:**
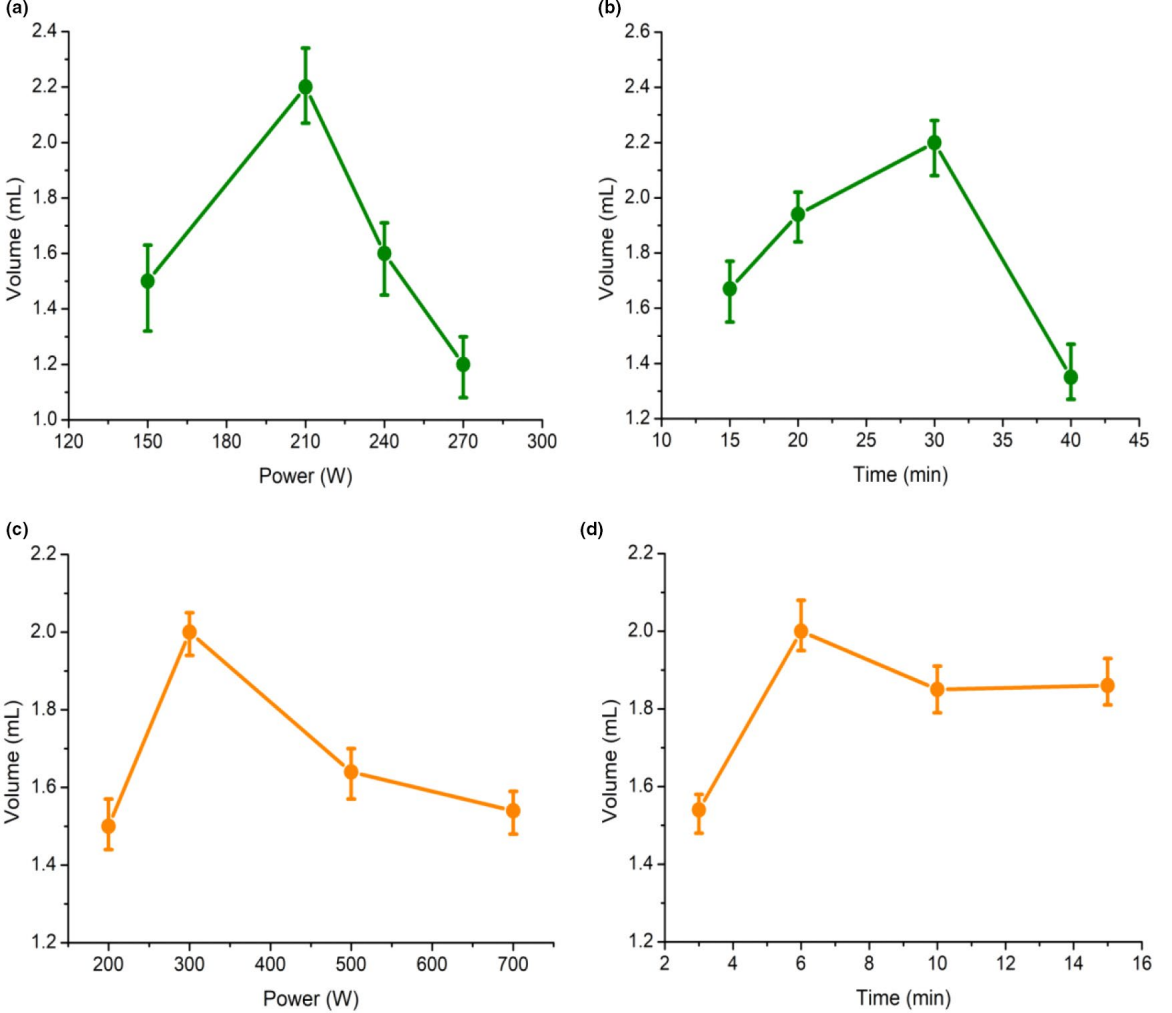
Single factor results for different extraction methods. (a) Effect of ultrasonic power on extraction yield; (b) effect of ultrasonic treatment time on extraction yield; (c) effect of microwave power on extraction yield; and (d) effect of microwave treatment time on extraction yield

As can be seen from Figure 1a,b, both ultrasonic power and ultrasonic time had significant influence on the extraction yield of EO. Corresponding to the increase of ultrasonic power and ultrasonic time, the volume of EO first increased and then decreased. As recognized, ultrasound is widely used for extraction of various substances from plant material and this generates microscopic bubbles. Under suitable ultrasonic power and time conditions, the collapsing bubbles are believed to create high‐shear gradients by causing microstreaming that disrupts the cell walls. This significantly accelerates the penetration of solvent into cells and the release of components from cells into the solvent, and simultaneously significantly enhances the mass transfer rate, further increasing the extraction yield (Tian et al., 2013). Figure 1a,b show that an excessively long ultrasonic time and a high power both had a negative effect on the yield. This result is consistent with previous studies (Goula, 2013; Zhang et al., 2008). This may be attributed to more cell walls being ruptured owing to a longer ultrasonic time and higher power, leading to impurities such as insoluble substances and cytosol being suspending in the extract, lowering the permeability of solvent into cell structures, and reducing the transfer of dissolved oil out of the solid structure (Tian et al., 2013), all of which causes decreased extraction yield. In summary, the optimal conditions for UP are operating at 210 W of ultrasonic power and an ultrasonic time of 30 min.

#### 3.1.2 Effect of Microwave power and microwave time on the yield

It can be seen that microwave has a membrane‐breaking effect on kumquat peel cells (Figure 4d), which contributes to extracting the EO from the kumquat peel oil cells (Allaf et al., 2013). According to Figure 1c, the extraction yield increased significantly before the microwave power reached 300 W and then subsequently decreased. The positive influence of the microwave irradiation on extraction yield can be attributed to the accelerated destruction of the plant cells followed by the rapid diffusion rate of intracellular constituents into the liquid solution (Akhbari et al., 2018). The reduction of the extraction yield of EO, on the other hand, is related to the rapid variations of temperature as a consequence of excessive microwave irradiation. The latter caused partial thermal decomposition of EO which had a detrimental effect on extraction yields (Chen et al., 2016; Liu et al., 2018). Irradiation time is also a factor studied to increase the effectiveness of extraction of EO. Studies were performed at different times. As can be seen from Figure 1d, with increasing the irradiation time from 1 to 6 min, the extraction yield of EO increased and reached its maximum at 6 min. However, the extraction yield decreased with this irradiation time. A possible reason may be due to the emulsification of EO at long irradiation time. Thus, 6 min considered as the appropriate irradiation time.

#### 3.1.3 Effect of different drying methods on the yield

**FIGURE 2:**
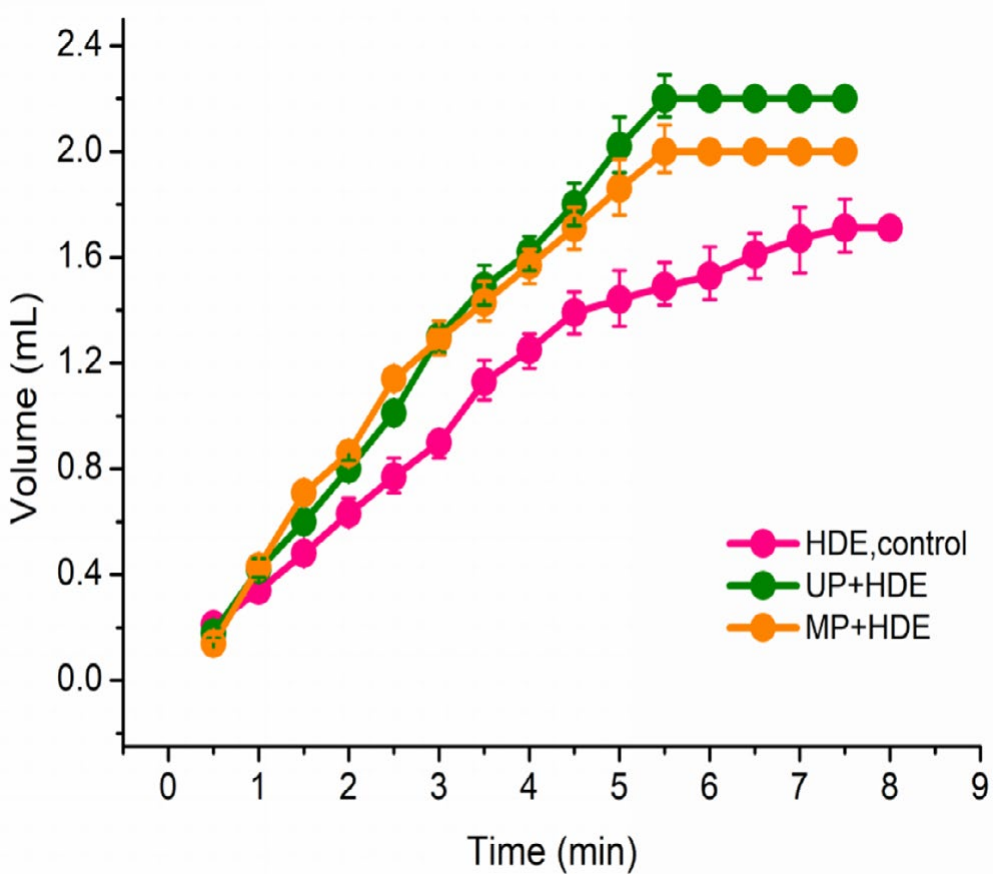
Extracting kinetics of kumquat Peel essential oil extracted by three extraction methods. HDE, hydrodistillation extraction; UP, ultrasound pretreatment; MP, microwave pretreatment

One of the main purposes of this research was to evaluate these new extraction techniques based on the amount of EO obtained. According to Figure 2, UP and MP increased EO yield as compared with HDE individually. Similar result was observed in extraction of EO from Trichodesma africanumand (Jaradat et al., 2016). For achieving maximum extraction yield, 467 min were needed for HDE. This time reduced to 339 and 326 min by use of UP and MP, respectively, indicating that the two methods improve the extraction efficiency. These results could be due to that UP and MP caused changes in the cell structure (Figure 4), and thereby accelerated the release of EO from plant matrix into the medium to make the EO can be easily extracted. Comparing UP with MP, MP had a lower EO yield than UP, which might be due to its strong thermal effect caused the loss of EO.

### 3.2 Extraction kinetics

The changes in the volume (V) of EO extracted by each method are presented in Figure 2. Parameters of the model used to describe the extracting kinetics are summarized in Table 1. The values of *R* ^2^ were .9889, .9934, and .9951, respectively, and the error between the experimental values V_1_ and the predicted values V_1_ obtained from the model was small. This indicates that model with small modifications can describe the extraction behavior of kumquat peel EO accurately. As observed in Figure 2, in the initial stage of extraction, a rapid increase in oil volume was observed. However, as the extraction process progressed, the rate of oil distillation slowed down until the extraction rate reached a constant. This was most likely because EO tend to diffuse slowly from the undestroyed reservoir inside the plant particles to their surface in the latter stage of the process (Milojević et al., 2008). A similar trend has been reported in the literature for rosemary (Cassel et al., 2009) and aniseed (Romdhane & Tizaoui, 2005). Obviously, UP and MP enhanced the extraction kinetics in comparison with HDE individually. This was consistent with the *k* values of Table 1, showing that using ultrasound and microwave as a pretreatment has a positive effect on the extraction efficiency. Farhat et al. (2011) also reported one of the advantages of the microwave‐assisted extraction of EO was the improved extraction efficiency, which is also true for UAHE (Chemat et al., 2011; Morsy, 2016).

**TABLE 1:** Parameters of models describing the extracting kinetics of kumquat essential oil as affected by three extraction methods

Model	Extraction method	Parameters		The value of V_1_		Statistics	
		a	*k*	Experimental	Predicted	*RMSE*	*R* ^2^
$V_{t}=a‐e^{\left(V_{1}‐kt\right)}$	HDE	2.113	0.224	0.855	0.832	0.0024	.9889
	UAHE	2.699	0.310	1.100	1.146	0.0024	.9934
	MAHE	2.630	0.250	1.000	1.040	0.0015	.9951

Abbreviations: HDE, hydrodistillation extraction; MAHE: microwave‐assisted hydrodistillation extraction; *R* ^2^, coefficient of determination; *RMSE*, Root mean squared error; UAHE, ultrasound‐assisted hydrodistillation extraction.

### 3.3 Analysis of the chemical composition

The GC‐MS analysis was determined on the EO obtained under suitable extraction conditions. Table 2 shows that EO is primarily composed of several compounds. This result is somewhat different from the results reported by previous studies (Choi, 2005; Quijano & Pino, 2009) which showed that there are more than 20 components in kumquat peel EO. This may be related to the planting environment of the kumquat, the collection area, the collection time, and the pretreatment method (Turek & Stintzing, 2013). The main component of EO was d‐limonene, following by myrcene, which was consistent with previous studies (Koyasako and Science, 2010; Koyasako & Bernhard, 1983; Wang et al., 2012), and these two compounds are also the main active ingredients. On the other hand, the components of the EO obtained by three extraction processes were very similar to each other (Golmakani & Rezaei, 2010; Karakaya et al., 2014). This similarity indicates that HDE plays a decisive role on the EO composition throughout the extraction process.

**TABLE 2:** The components identified and their percentages obtained by three extraction methods

No	Compounds	Area (%)			RI^a^	RI^b^
		HDE	UAHE	MAHE		
1	Myrcene	1.74 ± 0.06^b^	1.68 ± 0.06^b^	1.79 ± 0.06^b^	991	990
2	d‐limonene	95.05 ± 0.25^a^	97.02 ± 0.06^a^	96.58 ± 0.06^a^	1,036	1,044
3	2‐decene	0.37 ± 0.02^d^	–	–	1,265	1,258
4	11‐Octadecenoic acid,(Z)‐ (8CI)	1.09 ± 0.07^c^	0.67 ± 0.06^c^	–	1953	1945
5	Dioctyl phthalate	–	–	1.64 ± 0.06^b^	2,387	2,394

Note

Different letters (a‐d) indicate significant differences at *p* < .05.

Abbreviations: RI^a^, retention indices were calculated using a homologous series of *n*‐alkanes (C8‐C40); RI^b^, literature retention indices.

### 3.4 Antioxidant activity

**FIGURE 3:**
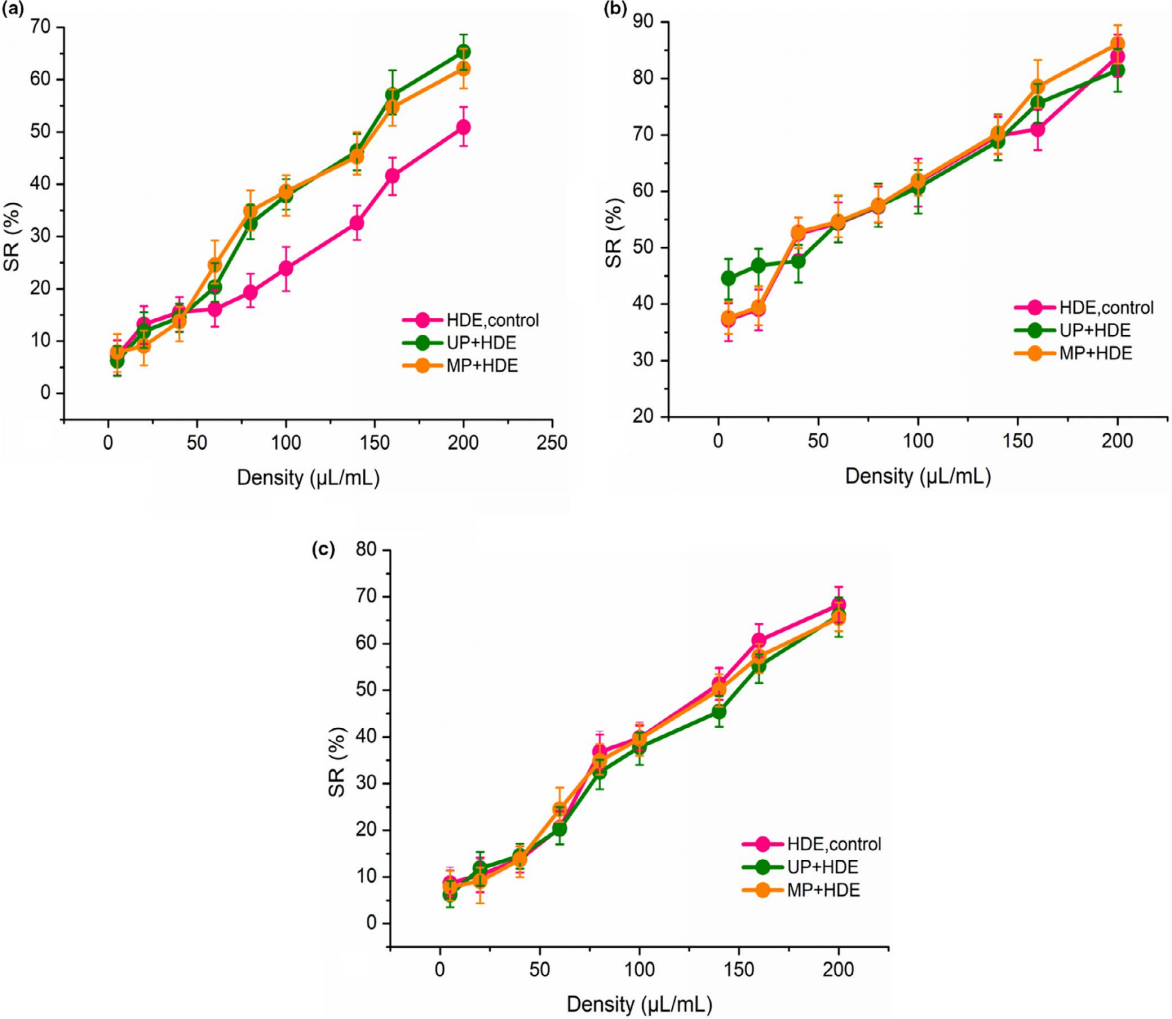
Comparison of (a) DPPH radical scavenging rate, (b) O2^−^ radical scavenging rate, and (c) ·OH radical scavenging rate of different concentrations of essential oil from three extraction methods. SR: scavenging rate; HDE: hydrodistillation extraction; UP, ultrasound pretreatment; MP, microwave pretreatment

Characterization of the antioxidant capacities of an EO should be performed using different assessment methodologies, as one oil can show a remarkable antioxidant activity with one methodology but have a poor activity profile with others (Graham, 1988). In this study, antioxidant activity of the EO from the kumquat peel was evaluated using DPPH, superoxide anion (O_2_^−^) radical, and hydroxyl radical (·OH). According to Figure 3, kumquat peel EO has a certain antioxidant ability. Moreover, UP and MP gave the EO a better antioxidant ability than HDE individually.

#### 3.4.1 Free radical‐scavenging activity (DPPH)

The DPPH is a stable free radical which can easily be reduced in the presence of an antioxidant, because most of the chemical ingredients in these natural antioxidants work synergistically with each other to produce a broad spectrum of antioxidative activities that creates an effective defense system against free radical attack (Singh et al., 2005). It can be obviously seen from Figure 3a that UP and MP gave the obtained EO a stronger capacities to scavenge DPPH free radicals than HDE individually, which may be strongly related to the type and quantity of EO components. As mentioned above, d‐limonene and myrcene, main components of EO, contain multiple unsaturated double bonds, they have a dynamic nature, strong hydrogen supply capacity and antioxidant effects. Moreover, the contents of these two components contained in EO obtained by UP and MP accounted for 98.7% and 98.37%, respectively, which were higher than that of HDE individually (96.79%).

#### 3.4.2 Superoxide anion and Hydroxyl radical‐scavenging activity

In Figure 3b,c, with increased EO concentrations, the scavenging capacity of hydroxyl radicals gradually increased, and it is seen that the EOs extracted by different methods have similar ability to scavenge superoxide anion radicals and hydroxyl radicals. The scavenging of superoxide anion and hydroxyl radical relies on chemically active components and their synergistic effect (Ennajar et al., 2015). Therefore, according to the similar types and contents of EO components (Table 2) from kumquat peel obtained by HDE with or without pretreatment, the above phenomenon can be well explained.

### 3.5 Structural changes after extraction

**FIGURE 4:**
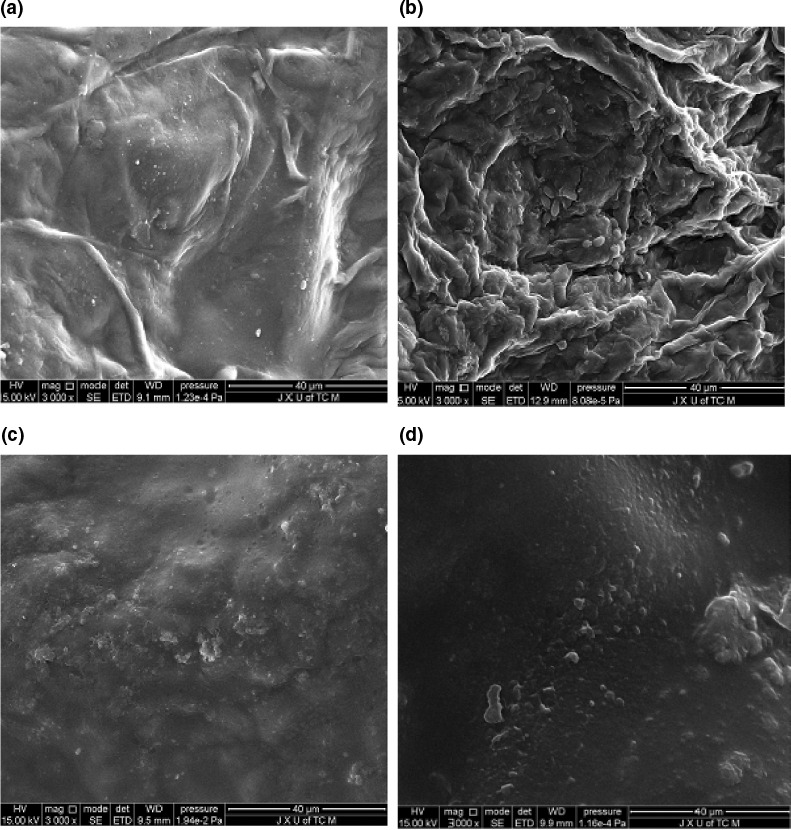
SEM images of (a) raw material, and residues obtained by (b) hydrodistillation extraction; (c) ultrasound‐assisted hydrodistillation extraction; (d) microwave‐assisted hydrodistillation extraction

Scanning electronic microscopy (SEM) was employed to evaluate the structural changes of kumquat peel when subjected to different oil extraction procedures. Figure 4a is a SEM image of the untreated kumquat peel (before extraction), and Figure 4b,d showed the micrographs of samples that had been treated by HDE individually and its combination with UP and MP, respectively. Compared with Figure 4a, the structure of plant treated with UAHE ruptured and formed porous structures. Similarly, the *Eletteria cardamomum Maton* showed a spongy, porous texture after ultrasound‐assisted extraction (Sereshti et al., 2012). It is well known that ultrasonic cavitation directly affects the texture and combines with the high temperature generated during the steam extraction process to form a porous structure and improve the effectiveness of solutes such as EO. In the MP process, heat transfer is mainly carried out by convection, conduction, and radiation. When the glands are subjected to more severe thermal stresses and localized high pressures, as in the case of microwave heating, the pressure build‐up within the glands could have exceeded their capacity for expansion and cause their rupture more rapidly than conventional extraction (Lucchesi et al., 2007). As shown in Figure 4f, the cells take on a puffy shape and collapse, some breaking in the process, and this is consistent with the results reported in relevant literature (Su et al., 2019). Therefore, efficient cell division is considered to be an important factor to improve extraction efficiency.

## 4 CONCLUSION

Both UP and MP increased the extraction yield and kinetics and DPPH scavenging activity of Kumquat peel essential oil (EO) obtained by hydrodistillation extraction (HDE), but did not noticeably affect chemical composition. In comparison with MP, UP gave a higher yield and DPPH radical‐scavenging activity of the EO. Overall, UP and MP possess the potential to be used in the HDE of EO from kumquat peel.

## CONFLICT OF INTEREST

It is declared that there is no conflict of interest in publication of this work.

## ETHICAL APPROVAL

Our research did not contain any animal experiments and human subjects.
